# Hereditary hemorrhagic telangiectasia associated with cortical development malformation due to a start loss mutation in ENG

**DOI:** 10.1186/s12883-020-01890-2

**Published:** 2020-08-26

**Authors:** Davide Villa, Claudia Cinnante, Gloria Valcamonica, Giulia Manenti, Silvia Lanfranconi, Annalisa Colombi, Isabella Ghione, Maria Cristina Saetti, Mario D’Amico, Sara Bonato, Nereo Bresolin, Giacomo Pietro Comi, Dario Ronchi

**Affiliations:** 1 Dino Ferrari Centre, Neuroscience Section, Department of Pathophysiology and Transplantation (DEPT), University of Milan, Via Francesco Sforza 35, 20122 Milan, Italy; 2 Fondazione IRCCS Ca’ Granda Ospedale Maggiore Policlinico, Neuroradiology Unit, Milan, Italy; 3 Fondazione IRCCS Ca’ Granda Ospedale Maggiore Policlinico, Neurology Unit, Milan, Italy; 4 Fondazione IRCCS Ca’ Granda Ospedale Maggiore Policlinico, Unità di Radiologia, Milan, Italy; 5 Fondazione IRCCS Ca’ Granda Ospedale Maggiore Policlinico, Neuromuscular and Rare Diseases Unit, Milan, Italy

**Keywords:** Cerebrovascular disorders, ENG, Hereditary hemorrhagic telangiectasia, Stroke, Case report

## Abstract

**Background:**

Hereditary hemorrhagic telangiectasia (HHT), also known as Rendu-Osler-Weber syndrome, is a rare disorder characterized by recurrent epistaxis, telangiectasias and systemic arteriovenous malformations (AVMs). HHT is associated with mutations in genes encoding for proteins involved in endothelial homeostasis such as *ENG* (endoglin) and *ACVRL1* (activin receptor-like kinase-1).

**Case presentation:**

Here we describe a 22-year-old male presenting with a transient episode of slurred speech and left arm paresis. Brain MRI displayed polymicrogyria. A right-to-left shunt in absence of an atrial septum defect was noted. Chest CT revealed multiple pulmonary AVMs, likely causing paradoxical embolism manifesting as a transient ischemic attack.

The heterozygous *ENG* variant, c.3G > A (p.Met1lle), was detected in the patient. This variant was also found in patient’s mother and in his younger brother who displayed cortical dysplasia type 2.

**Conclusions:**

The detection of cortical development malformations in multiple subjects from the same pedigree may expand the phenotypic features of ENG-related HHT patients. We suggest considering HHT in young patients presenting with acute cerebral ischemic events of unknown origin.

## Background

Hereditary hemorrhagic telangiectasia (HHT, ORPHA774) or Rendu–Osler–Weber syndrome, is a rare vascular disorder characterized by telangiectasias and arteriovenous malformations (AVMs) of skin, mucosae and internal organs [1]. The incidence, 1:5000–1:8000 worldwide, is likely underestimated because of the reduced age-related penetrance and variable clinical expression. Pulmonary and cerebral AVMs have been detected in 24–40% [2, 3] and 10–20% [4] of HHT patients, respectively. Although AVMs often remain clinically silent, cerebral AVMs rupture may cause intracerebral hemorrhage resulting in increased morbidity and mortality. Moreover, HHT patients may experience ischemic stroke or cerebral abscess because of paradoxical embolism due to the right-to-left shunting associated with pulmonary AVMs.

Clinical diagnosis can be achieved according to Curaçao criteria: 1) spontaneous and recurrent epistaxis; 2) multiple telangiectasia affecting lips, fingers, and nose; 3) telangiectasias and AVMs in visceral organs or central nervous systems and 4) a positive family history (first-degree relative) of HHT. A clinical diagnosis of HHT can be made when three of these criteria are met [5].

In most HHT patients, germline mutations in *ENG* (HHT1: OMIM 187300), encoding the TGF-β transmembrane receptor endoglin or *ACVRL1* (HHT2: OMIM 600376) which codes for the activin receptor-like kinase-1, are found. In addition, a phenotype of HHT and Juvenile Polyposis syndrome (JP-HHT) combined is caused by mutations in *SMAD4* [6]. Inheritance is autosomal dominant and haploinsufficiency is accepted as the predominant pathogenetic model [7].

Cortical development malformations (cDM) have been described in HHT patients with neuroimaging with a prevalence of 12% [8]. Clinical manifestations of cDM include epilepsy, cognitive abnormalities and developmental delay but symptoms might be heterogenous reflecting the location and the extent of cDM. Therefore, cDM prevalence in HHT is likely underestimated, particularly in subclinical forms. Neuroimaging findings suggest a clear prevalence of polymicrogyria in HHT patients with cDM [8, 9].

Recently, Klostranec and colleagues disclosed the co-occurrence of polymicrogyria and cerebral AVMs in close spatial proximity in six HHT cases and suggested that functional impairment of endoglin might produce focal hypoperfusion during corticogenesis in utero, leading to impaired neuronal migration and cortical organization, resulting in polymicrogyria [9]. Hypoxia is also expected to prime the appearance of the second somatic mutation in endothelial cells of adjacent vessels, establishing the condition for the later appearance of cerebral AVMs whose morphology and dimension become radiologically evident by age.

Here we present a novel Italian family with 3 affected individuals in two generations in whom the clinical diagnosis of HHT was confirmed by the identification of the previously reported [10, 11] *ENG* c.3G > A substitution. Two of them displayed cDM.

## Case presentation

Local Ethics Committee approved the study. Written informed consent was obtained from all subjects.

A 22-year-old man with a past medical history of focal epilepsy secondary to polymicrogyria and recurrent epistaxis presented to the Emergency Department with sudden onset of transient dysarthria and left-sided weakness. Cerebral CT scan and CT angiography in the acute setting were normal and ECG was normal.

**Fig. 1:**
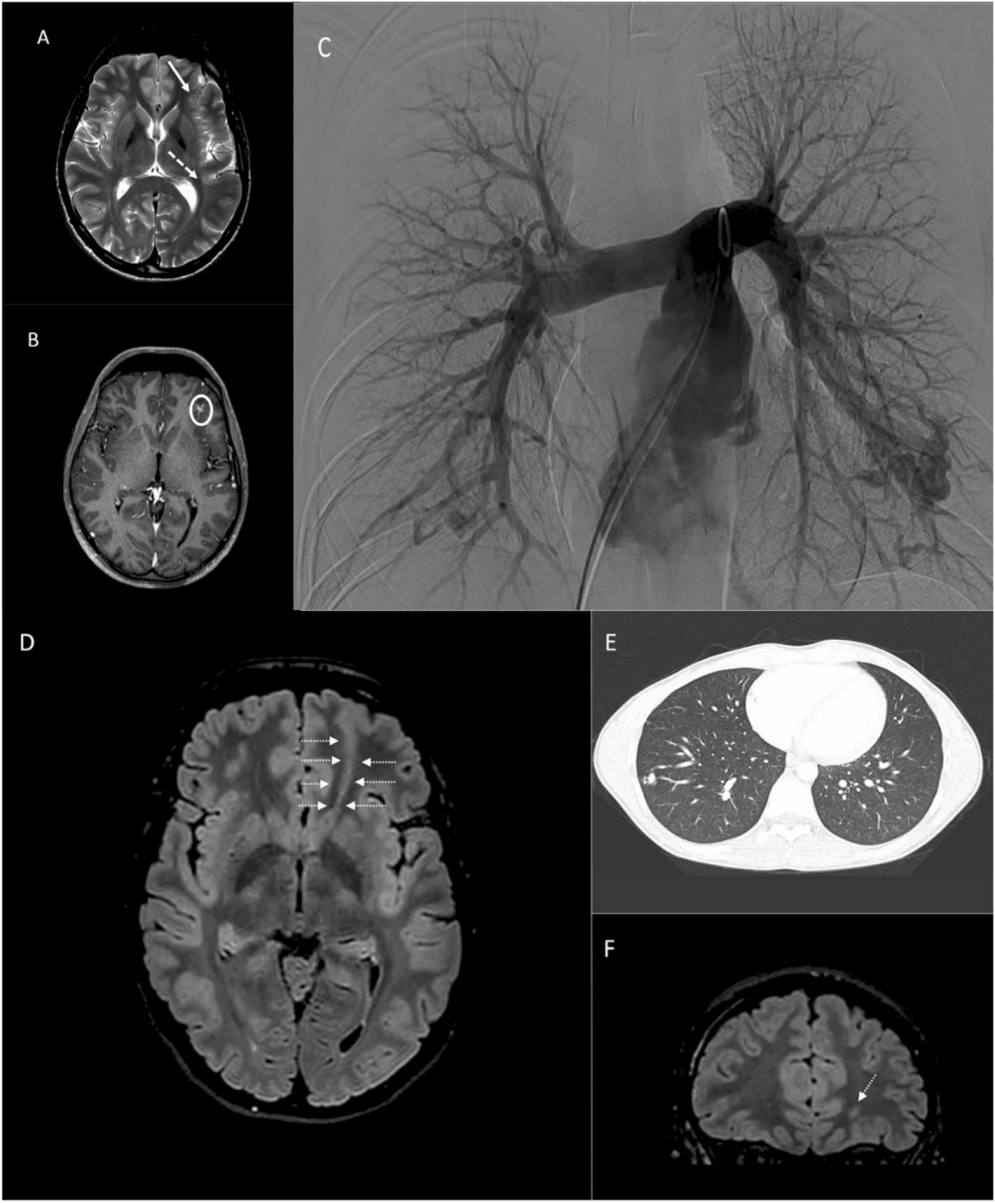
Neuroimaging findings in affected siblings (A-C: Proband, D-F: Probands’s brother). (**a-b**) MRI images (**a**: axial Turbo spin echo T2 sequence, **b**: axial post-gad 3D-T1 images) showing **a** a cortical development anomaly in left fronto-opercular frontal lobe (white arrow), where the cortex has a polymicrogyria; close to this cortical malformation, the post-gad T1 image **b** shows a small artero-venous shunt (white circle). In the left, posterior temporal lobe, axial T2 image **a** show an abnormal thickness of the cortex (white dotted arrow). **c** Digital angiography of pulmonary arteries shows multiple pulmonary arteriovenous malformations in the left lower lobe. **d-f** brain MRI Images **d**: axial Fluid attenuated inversion recovery FLAIR T2 image, **f**: coronal. FLAIR T2 image) showing a focal blurring in the left frontal lobe, between the medium and superior frontal gyrus, with a transmantle sign (white arrows) reaching the frontal horn of the lateral ventricle, as a cortical focal dysplasia, type II. **e** Chest CT study shows a pulmonary arteriovenous malformation in the right lower lobe

Brain MRI (Fig. 1a, b) confirmed left multifocal polymicrogyria and disclosed a right frontal acute stroke and multiple infratentorial and supratentorial AVMs. Spinal AVMs were absent but spinal MRI disclosed a focal cavity of the (cervical) ependymal canal. Transcranial ultrasound Doppler (UD) disclosed a right to left shunt with shower pattern. Carotid UD, cardiac echocardiography and blood tests including thrombophilia screening were normal. The transthoracic contrast-enhanced echocardiography revealed, however, an extra cardiac shunt. Chest CT was performed with evidence of multiple pulmonary AVMs as the obvious source of paradoxical embolism (Fig. 1c).

In the left lung the biggest AVMs measured 17 mm with three feeding arteries of 7, 3 and 2 mm in diameter, while in the right lung the biggest AVM measured 12 mm with a feeding artery of 2.5 mm in diameter.

Given the risk of a recurrent event, the patient was started on a full dose of low-molecular-weight heparin and underwent successfully endovascular embolization of all pulmonary AVMs. Anticoagulation was replaced by aspirin 100 mg daily six months later.

The patient’s mother had been clinically diagnosed with HHT with recurrent epistaxis and telangiectasias of the face and lips. She displayed pulmonary but no cerebral AVMs.

The patient’s younger brother (aged 20 years) suffered from recurrent epistaxis and was found to carry cortical dysplasia type II in the absence of cerebral AVMs (Fig. 1d, e) on cerebral MRI performed because of transient episodes of blurred vision. Pulmonary CT scan disclosed multifocal AVMs (Fig. 1f).

**Fig. 2:**
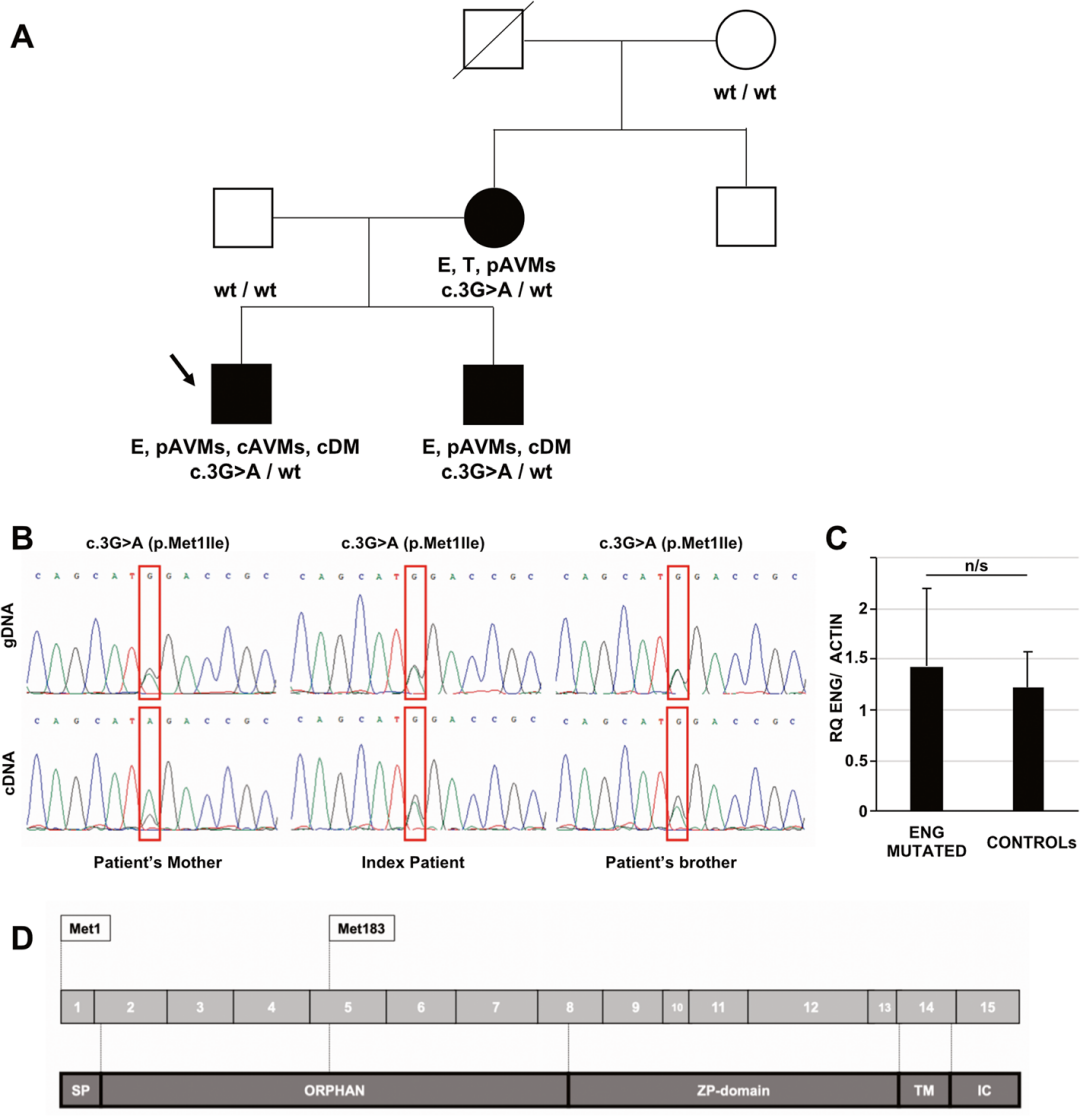
Molecular findings **a** Pedigree of the family described. Black arrow indicates index patient. Black symbols indicate affected subject. E: epistaxis, p/c AVMs: pulmonary/cerebral arteriovenous malformations, T: telangiectasias, cDM: cortical development malformation. Genotypes of available subjects are indicated below each symbol. **b** Molecular studies performed on genomic (gDNA) and complementary (cDNA) DNA in affected subjects. cDNA was obtained from blood-extracted RNA (Tempus Blood RNA isolation kit, Life Technologies) by retrotranscription (Maxima cDNA Synthesis Master Mix, Life Technologies). PCR and RT-PCR amplicons were analysed by direct sequencing: electropherograms show the nucleotide change c.3G > A in exon 1 of ENG. **c** Quantitative RT-PCR analysis of ENG transcript levels (probe: Hs.PT.58.4962347, IDT) in blood-extracted RNA from affected subjects of the study (ENG MUTATED, *n* = 3) and healthy subjects (*n* = 6). ACTIN (probe: Hs99999903_m1, Life Technologies) was used for normalization purpose. Determinations were performed in triplicate and Relative Quantification (RQ) levels were calculated by ΔΔCt method. n/s = not significant. **d** Scheme representing ENG coding exons (gray) and translated regions (dark gray). The position of physiological and alternative ATG codons are indicated

Coding regions and intronic boundaries of *ENG,* were analyzed by Sanger sequencing. A heterozygous *ENG* variant, c.3G > A (p.Met1lle), was detected in the patient. It was also found in the mother of the patient and in his younger brother who displayed cortical dysplasia type 2 (Fig. 2a). Complementary DNA was retrotranscribed by blood-extracted RNA and RT-PCR amplicons confirmed the expression of mutant allele in all affected family members (Fig. 2b). Quantitative probe-based RT-PCR studies did not show any difference in *ENG* transcript levels in mutated subjects compared to healthy controls (Fig. 2c).

## Discussion and conclusions

We report a familial case of HHT caused by the *ENG* heterozygous mutation c.3G > A (p.Met1lle). The mutation, predicted as pathogenetic by Mutation Taster, PolyPhen-2, SIFT and CADD, does not seem to impair *ENG* transcript stability, making haploinsufficiency unlikely as the driver mechanism of disease. According to NetStart [12] the mutation might cause the misstart of the protein at Methionine 183, resulting in the loss of the signal sequence and part of the orphan domain (Fig. 2d), which seems to play a role in ligand recognition.

The same change was previously described in familial cases of HHT [10, 11] without reported evidence of cortical malformations. In particular, the c.3G > A mutation was observed in 6 related patients in a cohort of 320 HHT subjects. Reported clinical findings included: epistaxis, telangiectasia, gastrointestinal bleeding and hepatic and pulmonary AVMs.

Cerebral AVMs are detected in about 10–20% of HHT patients [4]: they can cause cerebral hemorrhage but more frequently are clinically silent or underrecognized. Pulmonary AVMs allow blood to flow freely between the pulmonary and systemic systems without capillary bed filtering and may cause transient ischemic attacks or strokes. In our patient, transcranial Doppler ultrasound documented a right to left vascular shunt even in the absence of an atrial septum defect. Notably, pulmonary AVMs are more frequent in *ENG*-mutated subjects [13].

We observed cortical development malformation in two members of the family. In a published cohort of 116 HHT patients, authors identified cDM in 14 independent subjects (12%): 12 of them displayed polymicrogyria and 2 cases showed bifrontal periventricular nodular heterotopia [8]. Polymicrogyria was unilateral in all patients and had a perisylvian distribution. Clinical symptoms in these patients were epilepsy, hemiplegia, headaches, and stroke. AVMs were more frequently seen in cases with cDM. At least 5 of these cases harbored *ENG* mutations [8].

The findings in our index patient add support for an association of polymicrogyria to variants in *ENG* [9]. In particular, the finding of cerebral AVMs in close proximity with polymicrogyria cortex (left frontal and temporal-insular lobes) fits with the hypothesis that altered neuronal migration and cortical organization leading to polymicrogyria is the consequence of focal hypoperfusion during corticogenesis due to functional impairment of endoglin.

A novel finding of our report is the description of cortical dysplasia type II in patient’s younger brother. While spatial overlapping between cerebral AVMs and cortical dysplasia have been previously reported [14, 15], this is, to our knowledge, the first report of this type of cortical malformation in a HHT patient. Since cortical dysplasia was not reported in other mutated subjects of the family, we cannot exclude that this finding is an isolated event unrelated to *ENG* mutation and HHT.

Overall, our findings may expand the molecular and phenotypic features of *ENG*-mutated patients presenting HHT. Although HHT is relatively rare and literature data on risk of ischemic events due to pulmonary AVMs in HHT patients is still somewhat limited, we suggest considering HHT in young patients presenting with acute cerebral ischemic events of unknown origin, especially when the patient reports recurrent epistaxis.

## Data Availability

Not applicable.

## Abbreviations

HHT: Hereditary hemorrhagic telangiectasia
AVMs: Arteriovenous malformations
cDM: Cortical development malformations
MRI: Magnetic Resonance Imaging
ECG: Electrocardiogram
